# Recovery and decision-making involvement in people with severe mental illness from six countries: a prospective observational study

**DOI:** 10.1186/s12888-017-1207-4

**Published:** 2017-01-23

**Authors:** Sabine Loos, Eleanor Clarke, Harriet Jordan, Bernd Puschner, Andrea Fiorillo, Mario Luciano, Tibor Ivánka, Erzsébet Magyar, Malene Krogsgaard-Bording, Helle Østermark-Sørensen, Wulf Rössler, Wolfram Kawohl, Benjamin Mayer, Mike Slade, CEDAR Study Group

**Affiliations:** 1 Section Process-Outcome Research, Department of Psychiatry II, Ulm University, Ludwig-Heilmeyer-Str. 2, D-89312 Günzburg, Germany; 2 King’s College London, Institute of Psychiatry, Psychology and Neuroscience, London, UK; 3 Department of Psychiatry, University of Naples SUN, Naples, Italy; 4 Department of Psychiatry, University of Debrecen Medical Centre, Debrecen, Hungary; 5 Unit for Psychiatric Research, Aalborg Psychiatric Hospital, Aalborg University Hospital, Aalborg, Denmark; 6 Psychiatric Hospital, University of Zurich, Zurich, Switzerland; 7 Institute of Epidemiology and Medical Biometry, Ulm University, Ulm, Germany

**Keywords:** Clinical decision making, Patient involvement, Recovery, Severe mental illness (SMI), Routine mental health services, Multinational health service research

## Abstract

**Background:**

Clinical decision-making is the vehicle of health care provision, and level of involvement predicts implementation and satisfaction. The aim of this study was to investigate the impact of decision-making experience on recovery.

**Methods:**

Data derived from an observational cohort study “Clinical decision making and outcome in routine care for people with severe mental illness” (CEDAR). Adults (aged 18–60) meeting standardised criteria for severe mental illness were recruited from caseloads of outpatient and community mental health services in six European countries. After consenting, they were assessed using standardised measures of decision-making, clinical outcome and stage of recovery at baseline and 1 year later. Latent class analysis was used to identify course of recovery, and proportional odds models to investigate predictors of recovery stage and change.

**Results:**

Participants (*n* = 581) clustered into three stages of recovery at baseline: Moratorium (*N* = 115; 19.8%), Awareness/Preparation (*N* = 145; 25.0%) and Rebuilding/Growth (*N* = 321; 55.2%). Higher stage was cross-sectionally associated with being male, married, living alone or with parents, and having better patient-rated therapeutic alliance and fewer symptoms. The model accounted for 40% of the variance in stage of recovery. An increased chance of worse outcome (change over 1 year to lower stage of recovery) was found for patients with active involvement compared with either shared (OR = 1.84, 95% CI 1.15–2.94) or passive (OR = 1.71, 95% CI = 1.00–2.95) involvement. Overall, both process (therapeutic relationship) and outcome (symptomatology) are cross-sectionally associated with stage of recovery.

**Conclusions:**

Patient-rated decision-making involvement and change in stage of recovery are associated. Joint consideration of decision practise within the recovery process between patient and clinician is supposed to be a useful strategy to improve clinical practice (ISRCTN registry: ISRCTN75841675. Retrospectively registered 15 September 2010).

## Background

A policy consensus has emerged internationally supporting a mental health system orientation around recovery [1]. Converting this policy rhetoric into clinical practice has proved challenging, partly because the policy is not yet matched by a strong evidence base [2]. Syntheses are now beginning to be published addressing the concept of personal recovery [3] and its relation to outcome e.g. quality of life [4], specific pro-recovery interventions such as peer support [5], and implications for services [6]. However, wide variation is evident in emerging practices across different national and regional mental health systems [7]. The disparate commentaries on a recent overview [8] highlights the challenges of identifying best practice in supporting recovery.

More and different research is needed. Why different? Best available evidence indicates the key processes involved in recovery which are Connectedness (“community integration” in North America, “social inclusion” in the UK, continental Europe and Australia), Hope and optimism about the future, a positive non-stigmatised Identity, Meaning in life, and Empowerment—the CHIME Framework [3]. This framework has been validated internationally [9] and in current mental health service users [10], and the five processes are all potential target outcome domains for mental health services, yet the current evidence base and practice does not support this orientation. To illustrate this point, in England the National Institute for Health and Clinical Excellence (NICE) produces clinical guidelines for a range of disorders, including the schizophrenia guideline updated in 2014 [11]. No clinical trial evidence with primary clinical end-points involving any of the CHIME processes was cited in the evidence summary. Evidence about the relationship between clinical practice and recovery support is needed.

The necessary scientific building blocks are becoming available. Recovery measures have been developed [12, 13], and trials with recovery outcomes as primary clinical end-points are being published [14, 15]. However, an evidence gap remains about the relationship between clinical processes and recovery. Specifically, there is an absence of empirical evidence about recovery and clinical decision-making—the important process of treatment planning jointly between clinician and patient.

Clinical decision-making is the primary vehicle of mental health service delivery. Three levels of patient involvement in decision-making have been described: informed, shared and passive [16]. Passive decision-making occurs when the clinician makes the decision for the patient. Informed (or active) decision-making occurs when the patient makes the decision, having received information from the clinician. Shared decision-making (SDM) is collaborative decision-making involving the sharing of information and expertise by both participants.

Shared decision-making in mental health is widely recommended [11], despite being under-researched. A recent systematic review identified only two randomised controlled trials investigating shared decision-making [17], and a Cochrane review identified that ‘*further research is urgently needed*’ [18]. Despite the limited research base, it is recommended that ‘*a shared decision making approach should be facilitated*’ in adult mental health services [11].

In this study, the determinants of stage of recovery were investigated, with a particular focus on the experience of involvement in clinical decision-making. The aims are to identify (1) the course of change in stage of recovery, (2) cross-sectional predictors of stage of recovery, and (3) predictors of 1-year change in the stage of recovery.

## Methods

### Design

The CEDAR Study is a naturalistic prospective longitudinal observational study with bimonthly assessments during a 12-month observation period [19]. The overall aim of the CEDAR Study is to assess the scope and quality of clinical decisions in the treatment of people with severe mental illness, and the impact of clinical decision making in routine care on patient outcome.

### Participants and procedure

A total of 588 participants were recruited from caseloads of outpatient and community mental health services at six centres throughout Europe: Aalborg (Denmark), Debrecen (Hungary), London (England), Naples (Italy), Ulm (Germany) and Zurich (Switzerland). Inclusion criteria: aged 18–60 years at intake; mental disorder of any kind as main diagnosis established by case notes or staff communication using SCID criteria; presence of severe mental illness (defined as Threshold Assessment Grid (TAG) [20] score of > =5 and illness duration > = 2 years); expected contact with mental health services (excluding inpatient services) during the time of study participation; sufficient command of the host country’s language; capable of giving informed consent. Exclusion criteria: main diagnosis of mental retardation, dementia, substance use or organic brain disorder; cognitive impairment severe enough to make it impossible to give meaningful information on study instruments; treatment by forensic psychiatric services. After complete description of the study to the subjects, written informed consent was obtained.

Clinical staff rated TAG to identify presence of severe mental illness, and eligible patients were approached to give informed consent. At baseline, patients nominated a clinician closely involved in their treatment, and both completed baseline measures. At 1-year follow-up, patients and staff completed all baseline measures again. Bi-monthly, patients and staff were asked independently about context, content and implementation of clinical decision making. They wrote down the most important decision made at their last meeting, A list of predefined topics with three possible responses (“not discussed”, “discussed, no decision made” and “discussed, decision made”) was presented to indicated what was discussed in general in the selected meeting. Patients most frequently indicated having discussed “medication”, whereas staff reported “symptoms” most frequently, the third most frequent topic was “family” for both.

Ethical committee approval was obtained in all sites. Quality assurance in data collection was maximised by thorough training sessions for all study workers conducted by experts prior to the start of data collection, with biannual booster trainings for study workers during the data collection period. The characteristics of the sample are shown in Table 1.

**Table 1:** Sociodemographic and clinical characteristics of patient participants (*N* = 581)

Study centre *(n, %)*		
Ulm London Naples Debrecen Aalborg Zurich	111 80 101 97 97 95	19.1 13.8 17.4 16.7 16.7 16.4
Gender (female) *(n, %)*	306	52.7
Age (years) *(M, SD)*	41.7	10.8
Married *(n, %)*	145	25.5
Ethnic group (White) *(n, %)*	549	94.5
Years in school *(M, SD)*	10.4	1.9
Living alone *(n, %)*	230	39.6
Paid or self-employed *(n, %)*	109	18.8
Receiving state benefits *(n, %)*	419	72.2
Illness duration (years) *(M, SD)*	12.5	9.3
Diagnosis *(n, %)*		
Psychotic disorder Mood disorder Other	264 198 119	45.4 34.1 20.5
TAG *(M, SD)*	7.5	2.2
GAF (*M, SD)*	49.2	10.9

*Abbreviations*: *M* mean, *SD* standard deviation, *TAG* Threshold Assessment Grid, *GAF* Global Assessment of Functioning

### Measures

All measures were used in the local language. Existing translations were used when available, otherwise the measure was translated using intensive forward and backward translation by experienced bilingual clinical researchers following common standards [21]. All total scores except for Clinical Decision-Making Involvement and Satisfaction (CDIS) and Camberwell Assessment of Need Short Appraisal Scale (CANSAS) were pro-rated where 80% of items were completed.

Diagnosis was established at baseline from casenotes by researcher-assessed Structured Clinical Interview for DSM-IV Axis I Disorders—Clinical Version (SCID) [22].

Three patient-rated measures: *1. Stages of Recovery Inventory (STORI)* is a 30-item assessment resulting in allocation to one of five stages of recovery [23]. Because the original study and three replication studies [24–26] found a 3-cluster solution better fitted the data, a summary score comprising three stages was used: a) Moratorium (withdrawal characterized by a profound sense of loss and hopelessness), b) Awakening/Preparation (emergence of hope and taking first steps to work on recovery skills), c) Rebuilding/Growth (from actively working towards a positive identity and goals to a full and meaningful life). *2. Outcome Questionnaire-45 (OQ-45.2)* is a 45-item measure which provides an index of mental health functioning, ranging from 0 (good outcome) to 180 [27]. Sub-scales are symptom distress (range 0 to 100), interpersonal relations (0 to 44) and social role (0 to 36). Psychometric properties are confirmed in many studies with high internal consistency (.90) and test-re-test reliability (.84 over 3-weeks [28]). *3. Manchester Short Assessment of Quality of Life (MANSA)* is a 16-item assessment of quality of life ranging from 1 (low quality of life) to 7 [29]. Correlations between subjective quality of life scores on MANSA and Lancashire Quality of Life Profile were 0.83 or higher, Cronbach’s alpha for satisfaction ratings was 0.74.

Three staff-rated measures: *1. Global Assessment of Functioning (GAF)* is a staff-rated one-item global measure of symptomatology and social functioning, ranging from 1 (worst) to 100 (best) [30]. *2. Health of the Nation Outcome Scale (HoNOS)* is a staff-rated 12-item assessment of social disability ranging from 48 (worst) to 0 (best) [31]. *3. Threshold Assessment Grid (TAG)* is a staff-rated seven-item measure of severity (comprising Safety, Risk and Needs/Disabilities) of mental illness ranging from 0 (low severity) to 24 (20), and a score of 5 or more indicates severity [20].

Four measures rated by staff and patients: *1. Clinical Decision-Making Involvement and Satisfaction (CDIS)* scale measures involvement and satisfaction experienced with a specific decision, with versions rated by the service user (CDIS-P) and staff (CDIS-S) [32]. The Satisfaction sub-scale ranges from 1 (low satisfaction) to 5, and the Involvement sub-scale has three categories: Active (patient made the decision), Shared (decision jointly made by staff and patient) and Passive (staff made the decision). Note therefore that staff-rated passive involvement indicates passive involvement by the service user, i.e. active staff involvement. *2. The Clinical Decision Making Style Scale (CDMS)* [33] measured preferences for decision making. Parallel patient (CDMS-P) and staff (CDMS-S) versions rated on a five-point Likert scale. CDMS sub-scales are: Participation in Decision Making (PD), and Information (IN). *3. Helping Alliance Scale (HAS)* measures therapeutic alliance, with versions rated by staff (HAS-S, five items) and patients (HAS-P, six items) both ranging from 0 (low therapeutic alliance) to 10 [34]. *4. Camberwell Assessment of Need Short Appraisal Scale (CANSAS)* measures the presence of a met or unmet need in 22 domains, with versions rated by staff (CANSAS-S) [35] and patients (CANSAS-P) [35, 36]. Three summary scores are produced: unmet need, met need and no need, each ranging from 0 (low) to 22.

### Data analysis

To meet aim 1 (Stages of recovery), baseline STORI data were analysed using latent class analysis (LCA) [37] to identify adequate number of courses of recovery. In LCA, the observed variation in the indicator variable (stage of recovery) is ascribed to unobserved variation in the sample. Inter-individual differences concerning item response are explained by the existence of sub-groups with distinct response patterns. To keep the number of estimated parameters of the model within a reasonable range, we pooled the six response categories of the 30 items of the original measure into two categories (“not true now” and “true now”). Initial modelling involved the estimation of a single latent growth curve model, followed by the addition of a series of unconditional models. Models are viewed and compared based on practical considerations and information criteria to determine the adequate number of latent classes. In this study the models were compared using Bayesian Information Criterion (BIC). Better fitting models show a small BIC.

To meet aims 2 (Predictors of recovery stage) and 3 (Predictors of change in recovery stage), proportional odds models [38] were used. This approach is an extension of logistic regression, and was used because of the ordinal structure of the dependent variable. A latent continuum is divided into sections, and thresholds indicate to which observed category of the dependent variable the latent value relates (one threshold less than categories). The dependent variable was coded as 1 = Moratorium, 2 = Awareness/Preparation, 3 = Rebuilding/Growth.

For aim 3, generalized estimating equations (GENMOD procedure in SAS) were used to estimate the association between 1-year change in recovery stage (1 = improved (by one or two stages), 2 = no change, 3 = deteriorated (by one or two stages)) and baseline recovery stage, baseline and 1-year follow-up clinical measures and sociodemographic predictor variables. Several models were fitted, and the ‘Quasi-likelihood under the Independence model Criterion’ (QIC) statistic was used to compare models, with smaller QIC indicating better fit [39]. Analyses were conducted using Mplus software package 6.1, SPSS 21 and SAS 9.2.

## Results

A total of 581 participants self-rated stage of recovery at baseline: 115 (19.8%) Moratorium, 145 (25.0%) Awareness and preparation, 321 (55.2%) Rebuilding and growth. At 1-year follow-up, 512 (88.1%) of the 581 re-rated stage of recovery: 50 (9.8%), 153 (29.9%) and 309 (60.4%) respectively.

Completers of both measurement points did not differ from non-completers (with only one completed measure) with respect to sex, age, ethnicity, years in school, duration of illness, TAG and OQ-45 at T0. Completers had a significantly higher functioning (GAF mean = 49.40 (SD = 10.49) vs. 46.68 (SD = 13.57); *t* = −9.97(556), *p* = 0.002).

### Stages of recovery

**Fig. 1:**
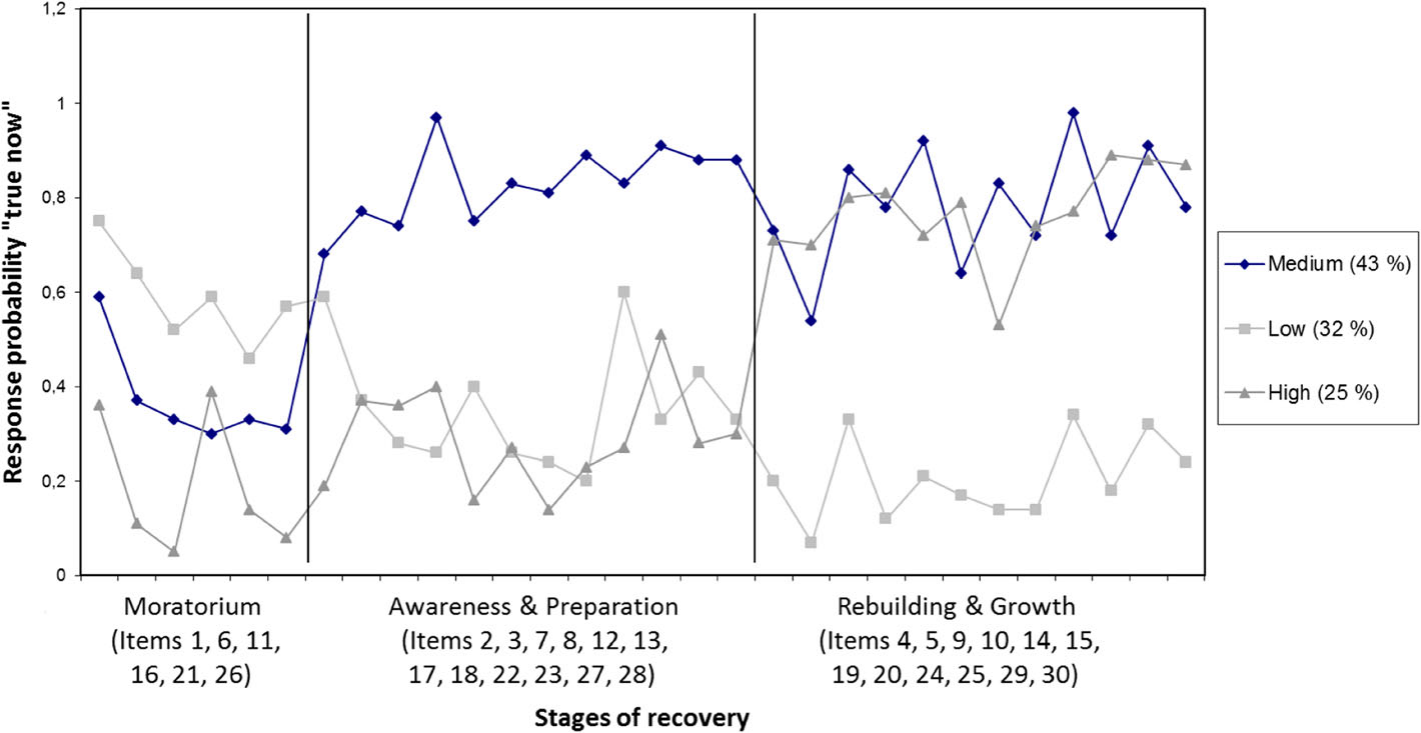
Latent class profiles for the three-class solution

We investigated the adequate number of courses of recovery by means of the number of classes representing a certain stage of recovery. Each stage of recovery maps toa certain response pattern of STORI items (e. g. being on a low recovery stage means high values in Moratorium items and lower values in other items). The latent class analysis of STORI data is shown in Fig. 1.

The three-stage model best fitted the data (compared to the five-class solution for recovery suggested by the original publication). This indicates that participants answers in the measure clearly cluster only into one of three stages of recovery, not in five (BIC = 21017.699). Class 1: medium stage of recovery, Class 2: lower stage of recovery, and Class 3: higher stage of recovery. The average probability for allocation into one distinctclass was 0.95 for class 1, 0.98 for class 2 and 0.97 for class 3.

### Predictors of recovery stage

The cross-sectional predictors of stage of recovery are shown in Table 2.

**Table 2:** Baseline predictors of stage of recovery (*n* = 397)

		B	SE	Wald	Sig.
Threshold					
STORI T0 = 1		−2.75	1.02	7.32	.01
STORI T0 = 2		−1.59	1.01	2.49	.12
Coefficients					
Centre (vs Zurich)	Ulm	.24	.29	.68	.41
	London	.21	.48	.19	.67
	Naples	−1.03	.37	7.80	**.01**
	Debrecen	−.23	.35	.45	.50
	Aalborg	.13	.34	.14	.71
Age		.01	.01	.82	.37
Gender (female vs. male)		−.42	.18	5.56	**.02**
Ethnicity (white vs. not)		−.49	.53	.86	.35
Years in school		.08	.04	3.76	.05
Married (yes vs. no)		.68	.29	5.56	**.02**
Living situation (alone vs other)		.68	.27	6.50	**.01**
Living situation (with parents vs other)		.86	.32	7.27	**.01**
Work status (employed vs not)		.31	.25	1.63	.20
State benefits (no vs. yes)		−.42	.27	2.46	.12
Duration of Illness		.00	.01	.07	.79
GAF		.01	.01	1.61	.21
HAS-P total		.10	.04	5.25	**.02**
OQ-45	Interpersonal relations	−.10	.02	37.65	**.00**
	Social role	−.08	.02	14.74	**.00**
CDMS	Participation	−.14	.15	1.41	.24
	Information	.02	.13	.02	.90
CDIS-P (vs Passive)	Shared	.13	.19	.44	.51
	Active	.41	.25	2.61	.11

Pseudo R^2^ = 0.40

*Abbreviations*: *STORI* stages of recovery inventory, *GAF* Global Assessment of Functioning, *HAS* Helping Alliance Scale, *OQ-45* outcome questionnaire, *CDMS* Clinical Decision Making Style Scale, *CDIS* Clinical Decision-Making Involvement and Satisfaction

Bold = *p* < 0.05

Higher stage of recovery was associated with being male, married, living alone / with parents (versus with others), not living in the Italian site (compared to those living in the Switzerland site), and having better patient-rated therapeutic alliance and fewer symptoms. The model accounted for 40% of the variance in stage of recovery.

### Predictors of change

At 1 year follow-up, 80 participants (15.8%) had deteriorated (changed to a lower STORI stage), 296 (58.5%) had no change, and 130 (25.7%) had improved (changed to a higher STORI stage). The best model (QIC fit index 1289.2; other models not shown) of predictors of change in stage of recovery is shown in Table 3.

**Table 3:** Generalized estimating equation for change in stage of recovery (*n* = 587)

	Odds ratio (95% Confidence interval)	*p*
Gender	0.77 (0.51–1.14)	.19
Ethnicity (White vs. nonWhite)	0.86 (0.25–2.97)	.82
Age	1.01 (0.99–1.03)	.48
Years in school	1.01 (0.90–1.13)	.88
Marital status (Married vs. not)	0.71 (0.44–1.12)	.16
Work status (Employed vs. not)	1.39 (0.85–2.24)	.19
OQ-total	0.99 (0.98–1.01)	.43
CANSAS-P unmet needs	1.01 (0.94–1.09)	.76
CDMS-P Participation	0.79−(0.60–1.04)	.10
CDMS-P Information	1.08 (0.82–1.44)	.58
CDIS-P Involvement (0 = active, 1 = shared)	1.84 (1.15–2.94)	**.01**
CDIS-P Involvement (0 = active, 1 = passive)	1.71 (1.00–2.95)	.05
CDIS-P Involvement (0 = shared, 1 = passive)	0.93 (0.65–1.34)	.69
MANSA	1.06 (0.82–1.29)	.77
HAS-P	0.99 (0.86–1.14)	.90
GAF	1.01 (0.99–1.03)	.19
HONOS	0.98 (0.95–1.01)	.25
Time (Baseline vs. 1 year)	1.19 (0.98–1.45	.08

Effects adjusted for all other appropriate effects in the model

*Abbreviations*: *OQ-45* outcome questionnaire, *CANSAS* Camberwell Assessment of Need Short Appraisal Scale, *CDMS* Clinical Decision Making Style Scale, *CDIS* Clinical Decision-Making Involvement and Satisfaction, *MANSA* Manchester Short Assessment of Quality of Life, *HAS* Helping Alliance Scale, *GAF* Global Assessment of Functioning, *HONOS* Health of the Nation Outcome Scale

Bold = *p* < 0.05

Only time-adjusted patient-rated decision involvement was significantly associated with a change in stage of recovery over 1 year. We found an increased chance for worse outcome (change to lower stage of recovery) for patients with active involvement compared with either shared or passive involvement. Patients who experienced active involvement compared to those with shared involvement had a 1.84-fold increased risk for worse outcome (*p* = .01), and compared to those with passive involvement had a 1.71-fold increased risk for worse outcome (*p* = .05). No significant interaction between time and involvement was found.

## Discussion

This multinational study on stages, predictors and change in recovery has three findings. First, empirical data distinguish between three distinct stages of recovery, which can be labelled as Moratorium (=cognitive, volitional and behavioral disengagement), Awareness/Preparation (=partial subjective engagement) and Rebuilding/Growth (=full behavioral engagement). The instrument STORI was originally based on a 5-category framework [36], and our study is consistent with three cluster-analytic studies [24–26] in identifying three distinct and interpretable stages. This indicates the need to develop treatment protocols which are organised by these stages of recovery.

Second, specific clinical, sociodemographic and geographic variables have cross-sectional association with stage of recovery. This adds more clarity to a comprehensive and multi-level evidence base for personal recovery. Compared to research into clinical recovery—the traditional understanding of recovery, involving sustained symptom amelioration and restoration of functioning—we already find a development of a comprehensive evidence base, including global epidemiological prevalence studies [36] and randomised controlled trial evidence investigating biological, psychological and social intervention.

Third, patients rating active involvement (compared with either passive or shared involvement in decision-making) at baseline were more likely to have changed to a lower stage of recovery 1 year later. This is a counterintuitive result at first glance. Experience by the patient of active involvement is influenced by role expectations, treatment context, information, and clinician behavior. There is emerging evidence that more active decision-making (even than initially preferred by the patient) is associated with increased satisfaction and subsequent decision implementation [40] and poorer involvement and satisfaction in regard to treatment-related decisions, compared with social and financial decisions [41]. Furthermore, a preference by clinicians for active rather than shared or passive patient involvement in decision-making is associated with reductions in patient-rated unmet need 1 year later [42].

One possible explanation would be that in the short term, active involvement is experienced as positive and empowering, whereas in the longer term (as in the current study) active involvement is a marker of staff-patient relationships which are insufficiently partnership-based. However, our therapeutic alliance measure (HAS) was not a significant predictor of change in stage of recovery. Therapeutic alliance was though a cross-sectional predictor of recovery, consistent with other empirical studies in which working alliance was a mediator of recovery [43].

The main strengths of the study are the large, varied, multisite sample recruited within routine mental health services. All patients were screened for severe mental illness, enhancing generalizability to other mental health systems. Multi-perspective assessments of decision-making by both staff and patients were used. In this naturalistic study, patients rated any type of decision they made with their clinician [41, 44], rather than the approach taken in some reviews [17] of restricting consideration to medical treatment decisions.

Several limitations apply. The use of a convenience rather than cohort sample in each site reduces representativeness, due to factors such as clinician bias in referral. More generally, optimal involvement in clinical decision-making may also differ between people with long-term mental health problems (as investigated here) and acute medical contexts. Measures used were patient and clinician self-report and did not include independent observer ratings of involvement style. The choice of predictors was a selective process and we cannot rule out that further variables might also influence the course of recovery.

## Conclusions

This study indicates that the relationship between involvement in decision-making and subsequent recovery is complex. The research implication of our study is that decision-making involvement and recovery are associated, so merit longitudinal investigation using standardised assessments to understand the direction of any causal relationship. It is plausible that the optimal level of involvement varies with stage of recovery. Therefore, clinical implication arising from this study to adapt patient involvement to changes in recovery process and preferences.

## Abbreviations

BIC: Bayesian Information Criterion
CANSAS: Camberwell Assessment of Need Short Appraisal Scale
CDIS: Clinical Decision-Making Involvement and Satisfaction
CDM: Clinical decision making
CDMS: The Clinical Decision Making Style Scale
CEDAR: Clinical decision making and outcome in routine care for people with severe mental illness
CHIME: Framework: connectedness; hope and optimism about the future; identity; meaning in life; and empowerment
GAF: Global Assessment of Functioning
GEE: Generalized estimating equations
HAS: Helping Alliance Scale
HoNOS: Health of the Nation Outcome Scale
LCA: Latent class analysis
MANSA: Manchester Short Assessment of Quality of Life
NICE: National Institute for Health and Clinical Excellence
OG-45: Outcome questionnaire-45
QIC: Quasi-likelihood under the Independence model Criterion
SCID: Structured Clinical Interview for DSM
SMI: Severe mental illness
STORI: Stages of recovery inventory
TAG: Threshold Assessment Grid
